# Role and Effectiveness of Intra-articular Injection of Hyaluronic Acid in the Treatment of Knee Osteoarthritis: A Systematic Review

**DOI:** 10.7759/cureus.24503

**Published:** 2022-04-26

**Authors:** Sumant Chavda, Syed Arman Rabbani, Tarun Wadhwa

**Affiliations:** 1 Orthopedics, Ras Al Khaimah (RAK) Medical and Health Sciences University, Ras Al Khaimah, ARE; 2 Pharmacology, Ras Al Khaimah (RAK) Medical and Health Sciences University, Ras Al Khaimah, ARE; 3 Clinical Pharmacy, Ras Al Khaimah (RAK) Medical and Health Sciences University, Ras Al Khaimah, ARE

**Keywords:** role and effectiveness, knee osteoarthritis, sodium hyaluronate, hyaluronic acid, intra-articular injection

## Abstract

Osteoarthritis (OA) is a degenerative joint disease that causes persistent joint pain and stiffness of mainly the large peripheral weight-bearing joints. It is a leading cause of functional disability and poor quality of life. Various modalities of therapy are recommended by different research organizations at different stages of OA including non-pharmacological, pharmacological, and surgical interventions. Intra-articular injections of hyaluronic acid (HA) is widely used for over three decades in the treatment of OA. However controversies exist regarding its safety and efficacy, the number of injections and courses, type of preparation, duration of its effects, and combining it with other drugs or molecules. This study aimed to review the most recent data available in the published literature to address these.

Electronic databases like Medline, Embase, ProQuest, and Google Scholar were searched for articles using keywords, intraarticular injections, hyaluronic acid, and osteoarthritis knee. The review was carried out as per PRISMA guidelines.

Thirty-eight randomized control trials (RCTs) investigating the efficacy and safety of intra-articular injection of HA were included in the systematic review. Out of the 38 studies, 22 (57.9%) were double-blind, eight (21%) single-blind, three (7.9%) non-blind, four (10%) with simple randomization, and one (2.7%) was open-labeled. Total 5,025 patients were included in these studies. The mean age of the patients was 60.28 years and the osteoarthritis grade of the knee joint was 1 to 3. HA was studied as a test preparation in 19 (50%) while in another 19 (50%) it was studied as a control. In 24 (63.2%) studies, HA was used as high molecular weight preparation in eight (21%) as low molecular weight preparation while in six studies the information was not available. HA was used as a standalone preparation in 31 studies, in two studies it was injected with platelet-rich plasma (PRP) and with either low-level laser therapy (LLLT), triamcinolone (TA), betamethasone (CS), poly deoxyribonucleotide (PDRN) or dexamethasone (DX) in one study each. In the majority of the studies, HA was given as a single injection (52.6% studies) or weekly three injections (28.9% studies). In 13.2 %, it was given as weekly 5 injections and in 5.3% as weekly two injections.

IA-HA injections have a limited role in the treatment of knee osteoarthritis in those patients who do not have sufficient pain relief with topical or oral medication and physical therapy. It is safe and effective except for minor side effects such as local pain and swelling lasting for a few days. Severe allergic reactions are extremely rare. They provide adequate pain relief and functional improvement for up to six months irrespective of a number of injections and type of preparations used. The combination formulations with corticosteroids or PRP or MSCs show better results than HA alone. Combining HA with newer molecules such as peptides or diclofenac for sustained and disease-modifying effects requires more studies in the future.

## Introduction and background

Osteoarthritis (OA) is a degenerative joint disease that most commonly affects the lower extremities’ large peripheral weight-bearing joints [1]. OA is one of the leading causes of functional impairment in the United States, affecting an estimated 22.7 million individuals [2]. It affects the quality of life by causing persistent pain, stiffness, and reduced mobility of the afflicted joints, as well as physical and/or mental co-morbidity [3]. It will boost healthcare spending, which is anticipated to be roughly $ 128 billion in the United States [4].

Because of its vast complexity and interplay of various biological factors such as genetic alterations, sex hormone deficit, and aging, OA is still poorly understood [5]. Many recent studies have focused on molecular markers that have been linked to chondrocyte senescence caused by stress [6]. Chondrosenescence is a term used to describe the age-related decline in chondrocyte function [7]. A core set of evidence-based therapeutic modalities has been created, according to the OA Research Society International (OARSI) Guidelines and recommendations for OA care [8]. These modalities include non-pharmacological approaches such as patient education and awareness, physical activity, and rehabilitation aids; as well as pharmacological modalities such as acetaminophen prescriptions, and non-selective NSAIDs (Nonsteroidal anti-inflammatory drugs), selective COX-2 inhibitors, and even opioid prescription. The most often recommended OA medications are NSAIDs [9]. Despite the fact that NSAIDs’, have been shown to be useful in the treatment of pain, long-term usage is linked to the risk of serious side effects. Furthermore, due to pharmacogenomics interactions, their tailored response is quite heterogenous [9].

Glucosamine and chondroitin, intra-articular injections of viscosupplements, corticosteroids, and platelet-rich plasma (PRP) are some of the additional non-operative treatment options [8]. Surprisingly, reduction in BMI, physical treatment, such as mind-body exercises, strength training activities, and aerobic workouts, have all showed promise in improving OA prognosis when patients follow their physical therapy routine regularly [10]. Table 1 outlines the current guidelines for OA treatment regimens from several international associations [11,12].

**Table 1:** Pharmacological and procedural recommendations for treatment of knee osteoarthritis NSAID=non-steroidal anti-inflammatory drug, I-CS=intra-articular corticosteroid, I-HA=intra-articular hyaluronic acid, SNRI=Serotonin and norepinephrine reuptake inhibitors, I-PRP=intra-articular platelet-rich plasma

Society	Recommended	Inconclusive	Not recommended
American Academy of Orthopedic Surgeons	Topical NSAIDs Oral NSAIDs Tramadol	Acetaminophen Nontramadol opioids I-CS I-PRP	Chondroitin Glucosamine I-HA
Osteoarthritis Research Society International	Topical NSAIDs Capsaicin Acetaminophen (in patients without comorbidities) Oral NSAIDs ( in patients without comorbidities) SNRI (i.e. duloxetine in patient without comorbidities) I-CS	Chondroitin (for symptom relief) Glucosamine (for symptom relief) Opioids I-HA	Chondroitin (for disease modification) Glucosamine (for disease modification)
American college of Rheumatology	Topical NSAIDs Acetaminophen Oral NSAIDs Tramadol I-CS	SNRIs (i.e. duloxetine) Nontramadol opioids I-HA	Topical capsaicin Glucosamine Chondroitin

In the last few decades, there has been an increasing trend to use intra-articular injections of corticosteroids, analgesics/anti-inflammatory drugs, polymerized collagen, anti-cytokine drugs, or hyaluronic acid as alternative modalities to maximize the local effect while minimizing systemic side effects [13]. Since its approval in Japan and Italy in 1987-1988, intra-articular (IA) injections of hyaluronic acid (HA) have been frequently utilized. Even after successful use of IA-HA for over three decades, controversies still exist regarding its safety, efficacy, number of injections, type of preparation, duration of its effects and combining it with other drugs or molecules. This systematic review aims to appraise the most recent clinical data available on I-HA therapy in patients with KOA. The objective of this systematic review is, 1) does I-HA has any role in the treatment of Knee OA? 2) is it safe and effective?

## Review

Methods

Study Design and Search Strategy

The review was performed as per the PRISMA guidelines. The search was carried out in the databases such as Medline, Embase ProQuest and Google scholar. Keywords like hyaluronic acid, hyaluronate injection, and osteoarthritis knee were used individually and in combination for the search.

Study Criteria

**Figure 1:**
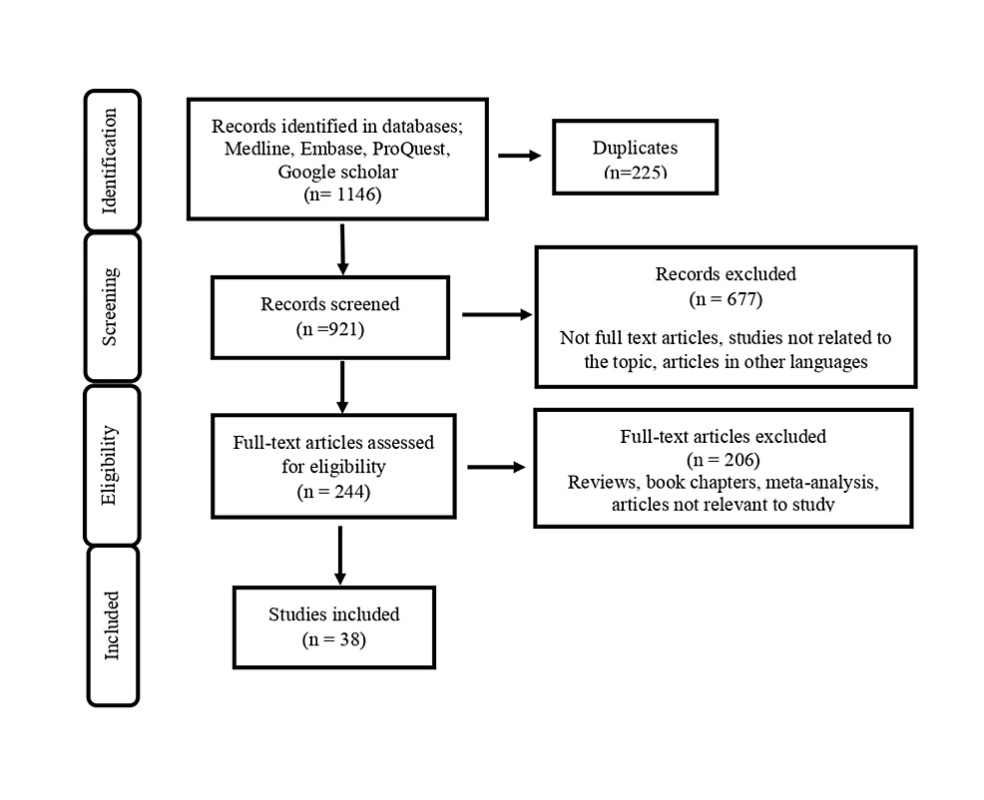
Study selection flow chart

All randomized control trials (RCTs) investigating the efficacy and safety on intra-articular injection of HA from January 2015 to December 2021 were included in the review. Articles published in languages other than English, review articles, meta-analyses, book chapters, and editorials were not included in the review. The study selection process is represented in Figure 1.

Search Results

The literature search yielded 1,146 articles. Out of which 225 articles removed, as they were duplicate articles. Remaining 921 articles screened further for appropriateness. Further 677 articles excluded from the study as, they were not relevant or not published in English language. Out of remaining 244 articles, 206 articles excluded from the study, as these were review articles or book chapters or meta-analysis. Thirty-eight studies met our inclusion criteria were included in the review. Quality assessment of the trials included in the review was done using Cochrane risk-of-bias tool for randomized trials (version 2).

Studies’ Characteristics

Out of the 38 studies, 22 (57.9%) were double blind; 8 (21%) single blind, 3 (7.9%) non-blind, 4 (10%) with simple randomization and 1 (2.7%) was open labelled (Table 2).

**Table 2:** Study distribution, patients and injections characteristics RCT-Randomized control trial, DB- Double blind, SB-single blind, NB-non blind, SR-simple randomization, OL-open labelled, KL- Kelly-green and Lawrence, LMW-Low molecular weight, HMW-High molecular weight, NA- not available, SP-study preparation

Studies								
		2015	2016	2017	2018	2019	2020	TOTAL
RCT-DB		3	5	4	6	4	0	22 (57.9%)
RCT-SB		0	0	2	0	3	3	08 (21%)
RCT-NB		0	1	1	1	0	0	03 (7.9%)
RCT-SR		0	0	2	0	1	1	04 (10.5%)
RCT-OL		0	0	0	0	1	0	01 (2.7%)
TOTAL		3	6	9	7	9	4	38 (100%)
Patients’ characteristics								
No. of patients		628	701	665	1300	1426	305	5025
Mean age (years)		62.5	61.4	60.85	58.28	59.44	59.25	60.28(mean)
OA grade (KL)		1-3	1-3	1-3	1-3	1-3	1-3	
Injections characteristics								
No. of injections	1	2	2	5	4	5	2	20 (56.6%)
	2	0	0	1	1	0	0	02 (5.3%)
	3	1	3	1	1	4	1	11 (28.9%)
	4	0	0	0	0	0	0	00 (0.0%)
	5	1	2	0	1	0	1	05 (13.2%)
HA preparations	LMW	1	2	1	1	1	2	08 (21%)
	HMW	2	4	5	4	8	1	24 (63.2%)
	NA	1	1	1	2	0	1	06 (15.8%)
HA used as	SP	4	3	4	2	4	2	19 (50%)
	Control	0	4	3	5	5	2	19 (50%)

Total 5,025 patients were included in these studies. The mean age of the patients was 60.28 years and osteoarthritis grade of the knee joint was 1 to 3 (Kelly-green-Lawrence grade) (Table 2).

Out of 38 studies, HA was studied as a test preparation in 19(50%) while in another 19(50%) it was studied as a control. In 24(63.2%) studies HA was used as high molecular weight preparation in eight (21%) as low molecular weight preparation while in six studies the information was not available. HA was used as a standalone preparation in 31 studies, in two studies it was injected with platelet rich plasma (PRP) and with either low-level laser therapy (LLLT), triamcinolone (TA), betamethasone (CS), poly deoxyribonucleotide (PDRN) or dexamethasone (DX) in one study each. In majority of the studies, HA was given as a single injection (52.6% studies) or weekly three injections (28.9% studies). In 13.2 %, it was given as weekly 5 injections and in 5.3% as weekly two injections (Table 2). The details of characteristics of studies included in the systematic review are represented in Table 2. 

The minimum follow up of the patients was six weeks and maximum of four years with an average of 26 months for majority of the studies. The WOMAC (Western Ontario and McMaster university osteoarthritis index) score was used as an outcome measure in all studies. The other outcome measures assessed in some studies were knee and osteoarthritis outcome system (KOOS), European quality of life scale (EUROQoL), Knee quality of life (KQoL), Lequesne knee index (LKI), stair climb test (SCT), knee society score (KSS), patient global assessment (PGA), patient reported outcome (PRO), Japanese osteoarthritis measure (JKOM), visual analog scale (VAS), and Health assessment questionnaire (HAQ).

All of the studies reportedly concluded that intra-articular injections of HA have resulted in clinical improvement over baseline pain, stiffness and function up to three to six months. The improvement was more rapid when HA was combined with corticosteroid and more sustained when combined with PRP or BM-MSC. The most common AEs reported were arthralgia, join swelling, joint stiffness and back pain. 

Discussion

Osteoarthritis (OA) is a degenerative joint disease that most commonly affects the lower extremities’ mainly large peripheral weight bearing joints. The current multimodal knowledge of OA incorporates trauma, mechanical forces, biochemical cartilage degradation, inflammation, and metabolic derangements in contrast to the idea that OA is just a degenerative process [14]. 

We may continue to target our treatments on stopping OA at the cause rather than relieving pain with analgesic, as we understand more about the pathophysiology of OA. Various evidence-based methods of therapy, including non-pharmacological, pharmacological and surgical are advised by various research bodies at various phases of osteoarthritis. In individuals, who do not react well to oral or topical medicine and physical therapy, intra-articular injectable treatments with corticosteroids, viscosupplements, platelet rich plasma (PRP) and stem cells are recommended. Intra articular HA has been utilized widely to treat symptomatic knee OA over three decades. 

Hyaluronic acid physiology in synovial fluid of joint

Hyaluronate is a high molecular weight, widely distributed substance found in the cartilage and synovial fluid. It’s made up of alternating N-acetyl d-glucosamine and d-glucuronic acid residues connected by ß(1-4) and ß(1-3) bonds and has a molecular mass of 6,500 to 10,900 kDa [15]. It functions as a lubricant, a scavenger for free radicals, and a regulator of cellular activity including protein binding. The endogenous HA in the joint depolymerizes from a high molecular weight (6,500-10,900 kDa) to a lower molecular weight (2,700-4,500 kDa) as OA progresses, reducing the mechanical and viscoelastic properties of the synovial fluid in the affected joint [15,16]. Exogenous HA injections have therefore been employed clinically to attenuate the macerated activities of OA patients’ depolymerized endogenous HA. Although the exogenous HA may not fully repair and replace the characteristics and activities of the synovial fluid’s depolymerized endogenous HA, it may provide enough pain relief through a variety of methods [16]. Synthesis of proteoglycan and/or glycosaminoglycan, anti-inflammatory action, and viscoelasticity maintenance are among these processes [16]. Exogenous HA has a varied reaction; one possible reason for the varying efficacy of HA therapies on OA patients is the levels of hyaluronidases in a patient’s synovial fluid. Hyaluronidases are a group of enzymes that degrade hyaluronic acid by cleaving the β(1-4) linkages of HA and fragmenting it into smaller fragments before degradation [17].

Hyaluronic acid and its preparations for treatment of OA

HA is given to OA patients in one of the two ways, by mouth or by local injection. Synvisc and Synvisc-One (Genzyme); Gel-One (Zimmer); Hyalgan (Fidia); Spartz FX (Bioventus); Orthovisc (Anika); Euflexxa (Savient); Monovisc (Anika Therapeutics) and Gel-Syn (Institute Biochimique SA) are some of the injectable HA formulations available for clinical usage [18]. They differ in many ways; mean molecular weight, molecular structure (linear, cross-linked or both), source (animal versus bacterial bio-fermentation using modified organisms), method of crosslinking, volume of injection (0.5-6.0 mL), concentration (0.8-30 mg/mL) and posology [19]. For many years, animal source was regarded a conventional source; however, modified bacterial source is now the primary source since it is connected with cheaper costs and fewer adverse effects [20]. The pain relieving action of the injected HA is assumed to be produced through a number of different mechanisms. These include increasing extracellular matrix protein synthesis, modifying inflammatory mediators to prevent degradation, limiting lymphocyte motility, and preserving cartilage thickness, area and surface smoothness [21]. However, further study is needed to comprehend the physiological effect of HA.

Human clinical studies involving hyaluronic acid

Several clinical trials have developed numerous HA formulations and investigated their effectiveness and safety throughout the last decade. Despite the fact that several studies have shown that intra-articular HA injection can be a good non-surgical treatment for OA and can delay the need for joint replacement, there is still debate over its clinical efficacy and long-term viability.

Altman et al. concluded in 2004 that Durold in the affected joint duct with very high molecular weight HA(100,000 kDa) is not beneficial and has no superiority over placebo treated groups in terms of The Western Ontario and McMaster Universities Osteoarthritis Index (WOMAC) score and other efficacy parameters [22]. There is a wide range of responses to different injectable HA preparations; Johan et al. discovered that using of high molecular weight I-HA preparations resulted in clinically significant pain reduction [22]. In patients with knee OA, Lundsgaard et al. found no significant difference between the intra-articular injection of HA (Hylagan®; 6000 kDa) and injection of physiological saline [23]. When compared to I-CS, which offered the highest pain reduction during first four weeks after injection, Rodriquez-Merchan found that when compared to I-CS, which provided the most pain relief during the first 4 weeks post-injection, I-HA produced superior results at five and thirteen weeks, and that the enhanced impact lasted until 26 weeks [24]. Knee OA appears to be treated well and safely with repeated rounds of I-HA. The most prevalent adverse effect of repeated I-HA was joint swelling and arthralgia, according to a 2018 systemic evaluation of patients who had recurrent cycles of I-HA for up to 25 months [25].

Timing and duration of injection

One of the most contentious aspects of HA injection is when and how long it should be given, and if this has an influence on its efficacy and long-term viability. Cubukuc et al. compared Hylan G-F 20 and saline intra-articular injections three times a week in OA patients. They found that the HA group experienced optimal pain alleviation as early as the third week, and that functional improvement occurred by the eighth week [26]. Patrella et al. conducted a randomized controlled trial in 2006 to compare the effects of three vs six consecutive weekly HA injections. They showed that there are no variations in pain, function or patient satisfaction between 3 and 6 HA injections [27]. Huskisson et al. demonstrated in 1999 that 5 weekly intra-articular injections of sodium hyaluronate (Hyalgan A) resulted in a 6-month clinical improvement [28]. Another randomized controlled clinical trial found that that 5 weekly IA injections of HA (Hyalgan) in patients with knee OA offered long-term pain relief and improved patient function, and were at least as effective as continuous naproxen therapy for 26 weeks with fewer side effects [29].

Despite its success in KOA management, I-HA is contested, and there is no consensus among international recommendations on how to utilize it. We have tried to find the answer to these controversies by reviewing the most recent data of randomized controlled clinical trials of the last five years reporting the safety and efficacy of I-HA in KOA.

Efficacy and Safety

These clinical studies have investigated the safety and efficacy of I-HA in general [30-32].

and in different dose regimens of I-HA [33]. The preparations were used from various sources of I-HA - animal and non-animal [33,34] of low molecular weight (LMW) and high molecular weight (HMW) [35] of different structures - cross-linked and linear [36]. The different formulations of I-HA [37] were studied for safety and efficacy of I-HA in combination or in comparison with corticosteroids [37,38] collagen [39] bone marrow-derived mononuclear cells [40] umbilical cord-derived mesenchymal stromal cells [41] bone marrow mesenchymal stem cells [42] ozone therapy [43] polydeoxyribonucleotide [44] platelet-rich plasma [45,46] plasma rich in growth factor [47] amniotic suspension allograft [48] chondroitin [49] and N-acetyl cysteine [50]. All these trials conclude that I-HA is both safe and effective when used alone and in conjunction with other viscosupplements. Furthermore, they support I-HA’s therapeutic efficacy in reducing pain and improving function in patients with KOA. In trials comparing the safety and efficacy of I-HA to other viscosupplements, the vast majority of the studies found I-HA to be non-inferior to its competitors.

Single/Multiple Injections

A variety of I-HA formulations are currently on the market for the treatment of KOA. These preparations differ in origin, structure, molecular weights, concentrations and injected volume. The majority of I-HA preparations on the market today are multiple injection regimens; however, single injection I-HA has also been developed and is becoming more widely used in clinical practice. Zhang H et al. examined the effects of single and multiple injections of I-HA in KOA in a randomized multicenter study and found that single injections were non-inferior to multiple injections of I-HA in alleviating pain, knee stiffness, and increasing physical function over 18 and 26 weeks [33]. Baron et al. reported similar results showing that a single I-HA injection was clinically effective in the treatment of KOA [31]. Ha et al. found that a single injection of cross-linked hyaluronate was non-inferior to multiple injections of linear high molecular hyaluronate in reducing weight-bearing pain in symptomatic KOA [36]. These results indicate that number of I-HA injections may not have a significant difference in terms of effectiveness of the therapy. 

HMW or LMW Preparations and Cross Linking

Over the years, several studies on I-HA with different molecular weights for the treatment of KOA have been published, with contradictory results, but favoring HMW HA. A similar study that compared the effectiveness of a HMW HA with a LMW HA preparation, found that both reduced pain, stiffness and function in patients with KOA in a similar way [35].

Another study comparing the efficacy of chemically cross-linked HA and avian-derived HA in KOA patients found that the chemically cross-linked HA was superior to avian-derived HA in lowering the VAS pain score and total Western Ontario and McMaster Universities Arthritis Index (WOMAC) score as well as in decreasing the Lequesne index score [34]. These findings imply that the source of I-HA may have an impact on the therapy’s efficacy.

Alone or in Combination With Other Drugs or Molecules

I-HA, alone or in conjunction with corticosteroids has been shown in studies to be safe and effective for the management of KOA. Petrella et al. showed that a single I-HA injection alone or in combination of triamcinolone was well tolerated and decreased pain associated with KOA in a prospective, multicenter, randomized study [37].

Maia et al. undertook another prospective randomized study to assess patient outcomes with intra-articular HA infiltration in conjunction with dexamethasone in treatment of KOA. The WOMAC overall score and sub scores for pain, stiffness, and function improved significantly with I-HA alone and in combination with dexamethasone. It also resulted in improvement of knee extensor and flexor strength [38].

In patients with KOA, a multicenter study testing the efficacy of a novel intra-articular combination of sodium hyaluronate- chondroitin sulfate found that the combination significantly improved pain severity and reduced analgesic use [49]. In a prospective, randomized research done by Yoon et al. combining intra-articular injection of polydeoxyribonucleotide with HA improved the efficiency of HA in enhancing knee joint functioning. All of these trials show that combination therapies with HA significantly enhances clinical outcomes in terms of pain and function in patients with KOA [44]. 

In Comparison With Other Injectable Therapies

In the majority of trials comparing I-HA to other viscosupplements in KOA, I-HA had similar results to its competitors. Martin et al. compared the safety and effectiveness of I-HA to MD-Knee, a collagen-containing product. The study’s findings revealed at three and six months after treatment, both I-HA and MD-knee groups had equal clinical outcomes in terms of improved function and pain reduction [39].

A six-month randomized clinical research comparing the benefits of ozone treatment with I-HA in KOA found that both groups saw equal reductions in total WOMAC score and improvements in pain, stiffness and function in both the groups [43]. A double-blind randomized controlled clinical trial comparing the intra-articular injections of Platelet-Rich Plasma (PRP) with HA in the treatment of KOA found that both arms saw similar pain and functional improvements [46]. Another study comparing the effectiveness of Plasma Rich in Growth Factor (PRGF) vs HA patients with symptomatic mild to moderate KOA found that both PRGF and HA, produced significant reductions in pain evaluated by VAS and significant improvement in WOMAC and Lequesne scores at six-month follow-up [47].

Ozcamdalli et al. evaluated intra-articular injections of HA and N-acetyl cysteine (NAC) in the treatment of KOA and found that both were equally efficient in reducing pain and enhancing function in KOA patients. In addition, combination preparations consisting of HA with diclofenac [51] and HA with polynucleotides have been found to significantly improve pain in patients with KOA [52]. All of these trials show that I-HA is not inferior to other viscosupplements in the treatment of KOA [50].

Duration of Symptomatic Relief and Disease Modifying Effect

Recently the disease modifying effects of I-HA in KOA have also been studied [30,53]. Henrotin et al. conducted an open labelled multicenter study to investigate the effect of I-HA on cartilage degradation biomarkers and found that I-HA had a favorable effect on type II collagen turnover and cartilage volume, indicating that I-HA may have a potential structure- modifying effect in KOA patients [30].

Limitations of this study

Studies included are of specific time period as we wanted to look for more recent data (Table 3). Studies published in languages other than English are not included. 

**Table 3:** Characteristics of studies included in the systematic review KOOS=knee and osteoarthritis outcome system, EUROQoL=European quality of life scale, KQoL=knee quality of life, LKI=Lequesne knee index, PKC=pain killer consumption, XLHA=cross linked hyaluronic acid, WBP=weight bearing pain, RCT-SR/DB/SB/OL/NB=randomized control trial simple randomization/double blind/single blind/open label/non-blinded, ESWT=extracorporeal shock wave therapy, SCT=stair climb test, KSS=knee society score, SLS=single limb stance, TUG=timed up and go, PGA=patient global assessment, PRO=patient reported outcomes, SANE=single assessment numerical evaluation, LP-PRP=leukocyte poor platelet-rich plasma, IKDC=international knee documentation committee, PGRF=PRP derived growth factor, JKOM=Japanese osteoarthritis measure, LMWHA=low molecular weight hyaluronic acid, IMWHA=intermediate molecular weight hyaluronic acid, HMWHA=high molecular weight hyaluronic acid, VAS=visual analog scale, WOMAC=western Ontario and McMaster university osteoarthritis index, HAQ=health assessment questionnaire, BM-MSC=bone marrow derived mesenchymal cells, UC-MSC=umbilical cord derived mesenchymal cells, LLLT=low level laserK therapy, PT=physical therapy, NAC=N-Acetyl Cysteine, DX=dexamethasone, TA=Triamcinolone Hexacetonide, ASA=amniotic suspension allograft

	Study Details			Patient’s Characteristics			Injection Characteristics				Outcome		
Sr.	Author	Year	Study type	Number	Mean age (Yrs.)	OA grade	HA brand	Molecular weight (KDa)	No. of injections	Comparison group	Outcome measures assessed	Follow up	Conclusion
1	Henrotin et al. [32]	2017	RCT-DB	66	65	2-3	Kartilage Cross (XLHA)	NA	1	Saline	Reduction in Coll2-1	90 days	Reduction in cartilage marker was significant
2	Zhang et al. [33]	2015	RCT-DB	349	60	2-3	Durolane	90,000	1	Artz (HA)- 5 inj. (620-1200 kDa)	WOMAC, GSA	26 wks.	Effective and non-inferior
3	Yang et al. [34]	2018	RCT-DB	258	64	2-3	HYA-JOINT plus (CCH)	NA	1	Hylan G-F-20 (6000 kDa)	WOMAC, SLS, TUG	3-6 months	Improvement. CCH group superior to ADH
4	Gigis et al. [35]	2016	RCT-DB	80	67	2-4	LMW	1,000-1,500	5	HMW/3 inj. (6,000-7,000)	VAS, WOMAC	3 mn-1 yr	Significant improvement & no difference in groups
5	Ha et al. [36]	2017	RCT-DB	266	62	1-3	XLHA (Hyruan Plus)	≥10,000	1	HMWHA (3 inj)	WBP, WOMAC	12 wks.	Effective and non-inferior, safe
6	Petrella et al. [37]	2015	RCT-DB	98	59	2-3	Hydros(Hydro-gel beads)	NA	1	Hydros-TA and Synvisc-one	WOMAC	26 wks.	Clinical improvement
7	Aguiar et al. [38]	2019	RCT-DB	44	57	1-2	Orthovisc	1,000-2,900	1	HA+DX, DX alone	WOMAC, Flx & Ext. strength	6 months	Significant improvement
8	Martin et al. [39]	2016	RCT-DB	60	69	2-3	Md-knee (collagen)	600-1200	5	Supartz (HA)	LKI, VAS, PKC	3-6 months	Significant improvement both groups
9	Goncars et al. [40]	2017	RCT-SR	56	58	2-3	BM-MSCs	800-1,500	1	GO-ON (HA -3 inj)	KOOS, KSS	3-12 months	Significant improvement more in BM-MSCs
10	Matas et al. [41]	2018	RCT-DB	26	55	2-3	UC-MSCs 2 dose (0-6 mn)	90,000	2	UC-MSCs-1 dose, Durolane	PGA, WOMAC	52 wks	Improvement in all group. Sustained up to 12 month in UC-MSCs-2
11	Espinosa [42]	2016	RCT-NB	30	60	2-4	BM-MSC-LD, BM-MSC-HD Plus HA	1,500-2,000	1	Hyalone (1,500-2,000 kDa)	VAS, WOMAC, MRI, X-ray	3-12 months	Clinical improvement. Sustained up to 12 mon in BM-HSC-HD group
12	Raeissadat et al. [43]	2018	RCT-DB	141	60	2-3	Hyalgan	500-730	3	Ozone sol.	VAS, WOMAC (Persian version)	6 months	Clinical improvement both groups. no diff
13	Yoon et al. [44]	2019	RCT-DB	30	65	2-3	HA+PDRN (polydeoxyriboneucleotide placenetex)	1500-2000	3	HA (Hylone plus)	VAS, WOMAC, KSS	6 months	Effective and combination can be considered for OA treatment
14	Raeissadat et al. [45]	2020	RCT-SB	102	58	2-3	PRGF (2 inj.- 3 wks. apart)	730	3	HA (Hyalgan)	VAS, WOMAC, ADL, LKI	6-12 months	Satisfactory improvement higher in PRGF group at 12 mon.
15	Montanez et al. [46]	2016	RCT-DB	53	61	1-3	PRP	600-1,200	3	Adant (HA)	VAS, KOOS, EUROQOL	3-6 months	Clinical improvement both groups
16	Raeissadat et al. [47]	2017	RCT-SB	69	59	2-3	PRGF	500-730	2	Hyalgan (HA-3 inj)	VAS, WOMAC, LKI	6 months	Equally effective
17	Farr et al. [48]	2019	RCT-SB	200	55	2-3	ASA (amniotic suspension allograft)	1,000-2,900	1	Monovisc (1,000-2,900), saline	PRO, KOOS, VAS, SANE	3-6 months	Improvement, greater in ASA group
18	Ozcamdalli et al. [50]	2017	RCT-SB	20	55	2-3	Hyalan G-F 20	6,000	1	NAC (1 inj)	VAS, WOMAC	6 wks.	Effective. NAC reduces cartilage degradation markers
19	Ip [54]	2015	RCT-DB	70	75	3	Hyalgan+LLLT	500-730	5	Saline+PT+ Sham light irrad	WOMAC	Mean 7 yrs.	Prolongs longevity of knee joint
20	Strand et al. [55]	2016	RCT-DB	350	61	1-3	Gel-200	NA	1	Retreatment after 13 wks.	WOMAC	26 wks.	Effective and safe
21	Paterson et al. [56]	2016	RCT-DB	23	51	2-3	PA-PRP	6,000	3	Synvisc-one	VAS, KOOS, KQoL	12 wks.	Improvement, more significant in PRP group
22	Cole et al. [57]	2017	RCT-DB	111	56	1-3	Synvisc	6,000	3	PRP	VAS, WOMAC, IKDC	24 wks.	Significant improvement both groups
23	Lana et al. [58]	2017	RCT-DB	105	61	1-3	Euflexxa (HMW)	2,400-3,600	3	HA+PRP	VAS, WOMAC	3-12 months	Improvement. More and sustained in HA+PRP group
24	Lee et al. [59]	2017	RCT-SR	61	68	2-3	ESWT (3 sessions)	3,000	3	HA	VAS, WOMAC, SCT	1-3	Significant improvement both group
25	Suppan et al. [60]	2017	RCT-NB	127	59	1-3	GO-ON (5mL)	800-1,500	1	GO-ON (HA -3 inj- 2.5ml)	WOMAC	3 months	Good efficacy, tolerability and safety
26	Yu et al. [61]	2018	RCT-DB	360	48	NA	PRP	NA	5	HA, PRP+HA, Placebo	WOMAC, Karnofsky perfo	52 wks. Post Trt.	Improvement significant in PRP+HA group
27	Lamo‑Espinosa et al. [62]	2018	RCT-NB LT	27	60	2-4	BM-MSCs-LD/HD +HA	1,500-2,000	1	HA alone (Hyalone)	VAS, WOMAC	12-48 months	Safe and feasible with long term clinical improvement
28	Hangody et al. [63]	2018	RCT-DB	368	58	1-3	Cingal (HA+TA)	1900	1	Monovisc (1000-2900 kDa), saline	PGA, WOMAC	26 WKS	Effective, immediate and LT relief with Cingal > 26 wks
29	Wang et al. [64]	2018	RCT-DB	120	63	2-4	HA+CS (betamethasone)	600-1,500	1	HMWHA	VAS, WOMAC	6 months	Improvement, rapid in HA+CS group
30	Buendia et al. [65]	2018	RCT-SR	106	56	1-2	LP-PRP-1	90,000	1	HA (Durolane), NSAID	VAS, WOMAC, MRI, X-RAY	6-12 months	Improvement better in LP-PRP group at 52 wks.
31	Hermans et al. [66]	2019	RCT-OL	156	54	1-3	Hylan (G-F 20)	6,000	3	UC (usual care)	KOOS, PGA	52 wks.	Effective
32	Maheu et al. [67]	2019	RCT-DB	292	67	1-3	Ostenil1 Plus	1,000-,2000	1	Hyalan G-F 20	WOMAC	6 months	Effective and non-inferior
33	Takamura et al. [68]	2019	RCT-SB	311	61	1-3	Gel-200 (XLHA)	>5,000	1	Saline	VAS, WOMAC	26 wks.	Effective and clinical improvement
34	Tavassoli et al. [69]	2019	RCT-SB	95	63	1-2	PRP-1	730	3	PRP-2, HA-3 (Hyalgan)	VAS, WOMAC	12 wks.	Improvement, PRP better than HA
35	Di Martino et al. [70]	2019	RCT-DB	192	57	1-3	PRP	3,200	3	Hylubrix (HA)	VAS, IKDC, EUROQoL	24 mn & Mean 64 mn	Effective, not superior in PRP group
36	Bahrami et al. [71]	2020	RCT-SB	90	56	2-3	HMWHA (Arthromac)	NA	1	LMWHA (3 inj.)	VAS, WOMAC, LKI	2-6 months	Remarkable improvement both group with no difference
37	Kesiktas et al [72]	2020	RCT-SR	54	56	2-4	Prostrolane (Peptide)	1700-2100	1	HA (Biometics), PRP	VAS, WOMAC, HAQ	3 months	Significant improvement, better in peptide group
38	Mochizuki et al. [73]	2020	RCT-SB	59	67	1-4	Artz (LMWHA)	620-1,200	5	Suvenyl (IMMWHA ) (1500-3900)	VAS, JKOM	6 wks.	Significant efficacy. No difference

## Conclusions

The IA-HA injections have a limited role in treatment of knee osteoarthritis. It is recommended to use in mild to moderate knee osteoarthritis in patients who do not have sufficient pain relief with topical or oral medication and physical therapy. Not used as first line therapy as it's expensive and has no data to shows improved efficacy. It is safe and effective except minor side effects such as local pain and swelling lasting for few days. Severe allergic reactions to the preparations are extremely are. They provide adequate pain relief and functional improvement but for limited period of time up to six months irrespective of number of injections and type of preparations used. The combination formulations with corticosteroids or PRP shows better results than HA alone. Combining HA with newer molecules such as peptides or diclofenac for sustained and disease-modifying effects, requires more studies in the future.
